# Radiofrequency ablation following first-line transarterial chemoembolization for patients with unresectable hepatocellular carcinoma beyond the Milan criteria

**DOI:** 10.1186/1471-230X-14-11

**Published:** 2014-01-10

**Authors:** Lan Zhang, Xin Yin, Yu-hong Gan, Bo-heng Zhang, Ju-bo Zhang, Yi Chen, Xiao-ying Xie, Ning-lin Ge, Yan-hong Wang, Sheng-long Ye, Zheng-gang Ren

**Affiliations:** 1 Liver Cancer Institute, Zhongshan Hospital, Fudan University, 136 Xue Yuan Road, Shanghai 20032, China; 2 Key Laboratory of Carcinogenesis and Cancer Invasion (Fudan University), Ministry of Education, 136 Xue Yuan Road, Shanghai 200032, China

**Keywords:** Hepatocellular carcinoma, Radiofrequency ablation, Transcatheter arterial chemoembolization, Milan criteria

## Abstract

**Background:**

Recent studies suggest that a combination of radiofrequency ablation (RFA) and transarterial chemoembolization (TACE) may have theoretical advantages over TACE alone for treatment of hepatocellular carcinoma (HCC). The purpose of this study was to evaluate the effectiveness and safety of radiofrequency ablation following first-line TACE treatment in the management of HCC beyond the Milan Criteria.

**Methods:**

Forty-five patients who consecutively underwent RFA following first-line TACE treatment for HCC beyond the Milan criteria were enrolled in this study. RFA was performed within 1–2 months after TACE treatment in patients who had incomplete necrotic tumor nodules. Primary effectiveness, complications, survival rates, and prognostic factors were evaluated retrospectively.

**Results:**

Complete ablation was achieved in 76.2% of the lesions according to 1-month follow-up computed tomography/magnetic resonance imaging evaluation. The mean follow-up period was 30.9 months (range 3–94 months). There were no major complications after RFA therapy. The median overall survival was 29 months (range 20–38 months), with 1-, 2-, and 3-year survival of 89%, 61%, and 43%, respectively. Multivariate analysis revealed that tumor diameter (P = 0.045, hazard ratio [HR] = 0.228, 95% confidence interval [CI]: 0.054-0.968) and pretreatment serum alpha-fetoprotein level (P = 0.024, HR = 2.239, 95% CI: 1.114-4.500) were independent predictors for long-term survival.

**Conclusions:**

HCC beyond the Milan criteria can be completely and safely ablated by radiofrequency ablation following first-line TACE treatment with a low rate of complications and favorable survival outcome. Further assessment of the survival benefits of combination treatment for HCCs beyond the Milan Criteria is warranted.

## Background

Hepatocellular carcinoma (HCC) is the third most common cause of cancer-related mortality worldwide and the second most common in China [1,2]. Despite increased early diagnosis of HCC as a result of surveillance of high-risk populations, only 30% of patients diagnosed with early stage HCC are candidates for curative therapies such as surgical resection or radiofrequency ablation [3]. Moreover, the prognosis of patients with advanced or unresectable HCC remains unsatisfactory due to a low response rate and short time to progression. According to current treatment guidelines, transcatheter arterial chemoembolization (TACE) has been established as the standard therapy for patients who are not eligible for curative therapies [4,5]. Although the survival benefit of TACE treatment has been proved in two randomized clinical trials [5,6], TACE has a primarily palliative effect and does not achieve complete tumor necrosis therefore tumor relapse after TACE is universal [7]. Additionally, repetitive TACE treatments might damage liver function reserve and decrease survival time. Consequently, new strategies are needed to improve the effectiveness of TACE treatment for HCC patients.

Percutaneous radiofrequency ablation (RFA) has been accepted as a curative therapy for small HCC, and can provide better local control of the disease than TACE treatment and a similar long-term survival to surgical resection. Previous studies have shown that RFA can achieve complete necrosis in more than 90% of small HCCs [8-10]. Nevertheless, the effectiveness of RFA treatment in patients with intermediate or large HCC is unsatisfactory, with a relatively low complete necrosis rate ranging from 29.0% to 70.0% even if overlapping ablation or repeated procedures are used [11-13]. Therefore, conventional RFA treatment was recommended only for patients with small HCCs confined to the Milan criteria [14].

Recent evidence has suggested that TACE combined with RFA may have a synergistic effect on ablation of HCC [15-17]. To date, few studies have investigated combination therapy of radiofrequency ablation following transarterial arterial chemoembolization for patients with unresectable HCC beyond the Milan criteria. The aim of this study was to evaluate the effectiveness and safety of such combination therapy for unresectable HCC.

## Methods

### Patient enrollment

This survey is a retrospective study in a single center. The TACE or RFA treatment procedures were according to our institutional standard treatment protocol at Zhongshan hospital, Fudan University. The Medical Ethics Committee of Shanghai Zhongshan Hospital provided ethics approval for this retrospective study. From February 2001 to November 2009, a total of 611 consecutive HCC patients with unresectable tumors were treated with RFA in the Liver Cancer Institute, Zhongshan Hospital. All patients had a prospectively established database covering patient demographics, the etiology of the underlying liver disease, tumor-related characteristics, serum biochemistries, and survival data. Patient selection was performed using the following inclusion criteria: (1) diagnosis of HCC confirmed pathologically or in accordance with American Association for the Study of Liver Diseases criteria [18]; (2) HCC beyond the Milan Criteria (single nodule with size ≤5 cm or three nodules or fewer with tumor size ≤3 cm) that was treated with TACE as the first-line therapy; (3) tumor nodules to be ablated should be clearly observed by ultrasound and there should be no contraindications of RFA therapy; (4) no portal vein involvement or distant metastasis; (5) liver function of Child-Pugh A-B; (6) complete clinicopathologic and follow-up data. Ultimately, 45 patients were included in this study. Patient characteristics are shown in Table 1.

**Table 1:** Baseline characteristics of patients treated with RFA after first-line treatment with TACE

**Characteristics**	**No. of patients (%)**
Age (years)	
<60	27 (60.0)
≥60	18 (40.0)
Child-Pugh classification	
A	45 (100.0)
B	0 (0.0)
C	0 (0.0)
Gender	
Male	39 (86.7)
Female	6 (13.3)
HBsAg	
Positive	36 (80.0)
Negative	9 (20.0)
Anti-HCV	
Positive	2 (4.4)
Negative	43 (95.6)
AFP	
<400 ng/ml	25 (55.6)
≥400 ng/ml	20 (44.4)
Ablation nodules	84
Single	20 (23.8)
Multiple	64 (76.2)
Tumor diameter with ablation	
≤5 cm	44 (52.4)
>5 cm	40 (47.6)

### TACE procedure

TACE was performed using a standard procedure. Briefly, hepatic arteriography was performed to identify the feeding artery of the liver tumor. The catheter, usually a 4 F or 5 F RH catheter (Cook Co., USA), was inserted into the tumor feeding artery as close as possible to the tumor. Microcatheters (Terumo Co., Japan) were used to catheterize the feeding artery if needed. Chemotherapeutic agents [1000 mg 5-fluoruracil (5-FU), 80 mg cisplatin] were slowly infused followed by 5–30 ml lipiodol with 10 mg mitomycin-C emulsion for embolization. The regimen of chemoembolization was adjusted according to liver function and peripheral leukocyte or platelet levels. Gelatin was administered afterwards for additional embolization in cases with large hypervascular tumors.

### RFA procedure

Before RFA treatment, a dynamic contrast computed tomograph (CT) or magnetic resonance imaging (MRI) was performed to evaluate the response after TACE and identify the target lesions to be treated with RFA. Disappearance of tumor enhancement in the arterial phase was considered complete necrosis and identified tumor nodules that were not targets for RFA. Only tumor nodules with contrast enhancement in arterial phase with CT or MRI evaluation were treated with RFA.

From February 2001 to September 2004, 16 patients were treated with RFA using the RFA 2000 (Radio-Therapeutic, USA) and a needle electrode with a 15-gauge insulated cannula and 10 hook-shaped expandable electrode tines with a diameter of 2.0 or 3.5 cm at expansion (Radio-Therapeutics,USA). From October 2004 to November 2009, 29 patients were treated with RFA using a 15-gauge multielectrode RF probe with an expansion of 2.0 or 3.5 cm (RITA, USA). The details of the treatment procedure are described in our previous studies [19]. Briefly, RFA was performed by a percutaneous procedure under conscious sedation and local anesthesia. The RFA electrode was inserted into the tumor nodules under the guidance of ultrasonography. Overlap ablation was allowed to cover the whole tumor nodule and achieve an adequate safety margin of 0.5-1.0 cm.

### Evaluation of ablation efficiency and follow-up

The ablation efficiency of RFA was evaluated after 1 month by dynamic contrast-enhanced CT or MRI. Complete ablation (complete response [CR]) was defined as complete devascularization of the lesions during the arterial phase and no appearance of new tumors at other liver sites. Patients were monitored every 2 months after RFA by serum alpha-fetoprotein (AFP) levels and abdominal ultrasonography. Patients with test results suggestive of recurrence or metastasis received additional computed tomography and/or magnetic resonance imaging. Patients with local incomplete ablation or residual disease in the remaining liver underwent a repeat RFA or chemoembolization.

### Statistical analysis

Quantitative variables were expressed as means ± standard deviation or as medians. Qualitative variables were presented as numbers and percentages. Student’s *t* test was used to compare quantitative variables and the χ^2^ test was used for qualitative variables. Overall survival (OS) was calculated from the date of TACE treatment to death or the last follow-up. Survival curves were constructed by the Kaplan-Meier method. Multivariate analysis was performed using the Cox proportional hazards models. A difference was considered significant for P <0.05.

## Results

### Efficiency of ablation

We evaluated a total of 147 tumor nodules in 45 patients (10 patients with single nodule; 11 with 2 nodules, 2 with 3 nodules, and 22 with 4–5 nodules). Among the 147 nodules, 84 were treated with the RFA procedure. The other 63 nodules were not treated with RFA because of complete necrosis presenting with complete lipiodol deposition and without artery contrast enhancement on CT or MRI after TACE treatment. For the ablated tumor lesions, 44 lesions were ≥50 mm and 40 lesions were <50 mm, with a median tumor diameter of 55.0 mm (range 20 mm to 80 mm) (Table 1).

**Figure 1:**
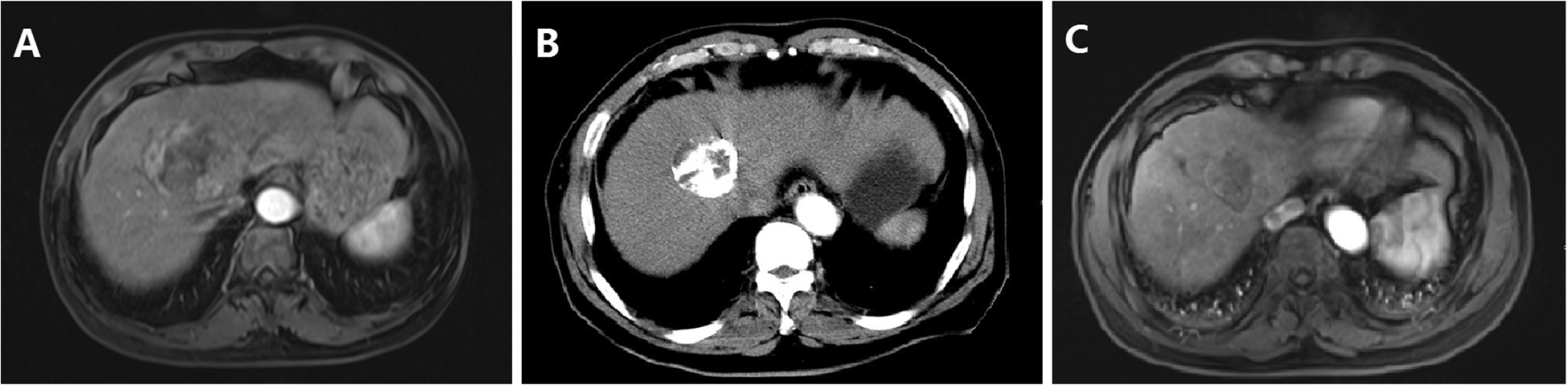
**Complete ablation of HCC. A**: MRI image before TACE; **B**: CT image before RFA; **C**: MRI image after RFA.

**Figure 2:**
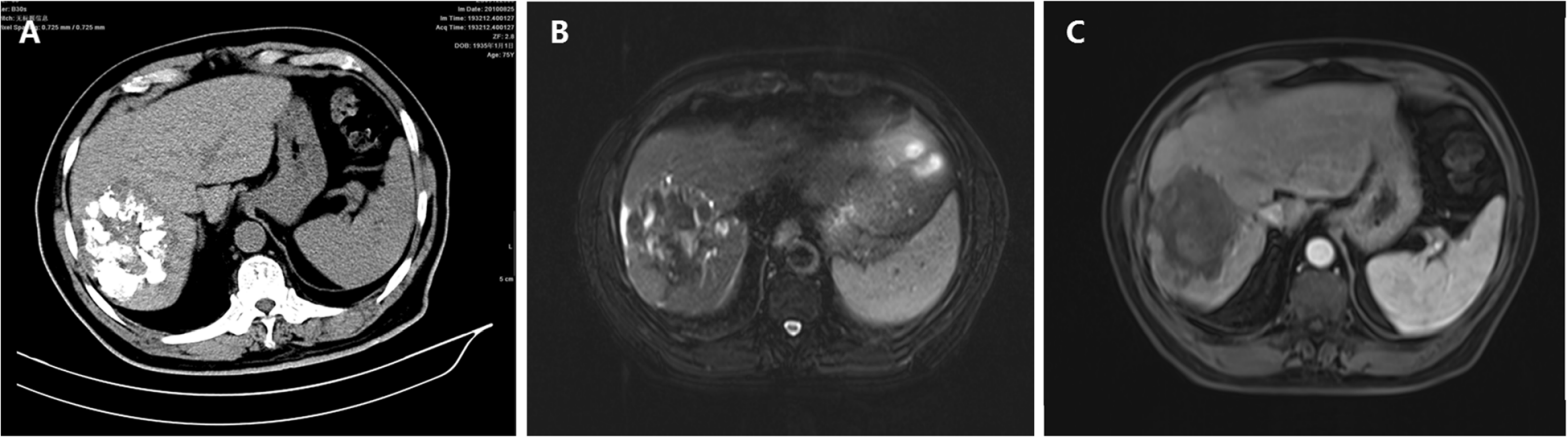
**Incomplete ablation of HCC. A**: CT image before RFA; **B** and **C**: MRI image after RFA.

Complete ablation was achieved in 64 of 84 (76.2%) lesions (Figure 1). The remaining 20 (23.8%) lesions were identified as residual disease attributed to incomplete ablation on CT/MRI images (Figure 2). The ablation efficiency showed a significant correlation with the size of the lesion: lesions <50 mm had a higher complete ablation rate than lesions ≥50 mm with complete ablation rates of 86.4% (38/44) and 65% (26/40), respectively (P = 0.022). However, the number of lesions had no impact on the complete ablation rate. Complete ablation was achieved in 51 of 64 (79.7%) lesions in the 25 patients with multifocal tumors, compared with 13 of 20 (65.0%) lesions in the 20 patients with a single lesion. There was no significant difference between patients with single or multiple nodules (P = 0.178) (Table 2).

**Table 2:** Evaluation of factors affecting ablation efficiency

**Prognostic factor**	**Local success (CR)**	**P value**
Tumor diameter		
≤5 cm	86.4% (38/44)	0.022
>5 cm	65.0% (26/40)	
Tumor number		
Single	65.0% (13/20)	0.178
Multiple	79.7% (51/64)	

### Survival

**Figure 3:**
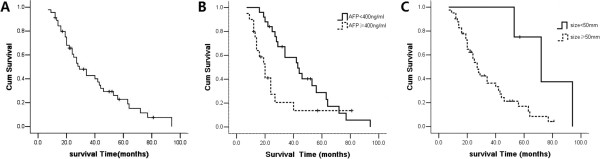
**Survival curves of patients treated with RFA after first-line treatment with TACE. A**: Overall cumulative survival of 45 patients treated with RFA after first-line treatment with TACE; **B**: Cumulative survival according to AFP level; **C**: Cumulative survival according to tumor size.

During a median follow-up of 30.9 months (range 3–94 months), 33 patients died from intrahepatic recurrence and three patients died from non-HCC related causes. The median OS was 29 months (range: 20–38 months) with 1-, 2- and 3-year overall survival rates of 89%, 61%, and 43%, respectively (Figure 3A).

### Complications

Two of 45 patients (4.4%) developed procedure-related major complications after RFA (one with local skin heat injury and one with gastrointestinal bleeding). No procedure-related deaths occurred. Minor complications such as nausea, fever, and post-treatment abdominal pain were observed in most patients but none required medical intervention.

### Prognostic factors

The prognostic factors used for univariate analysis included the variables of demographics, liver function, and tumor-related characteristics. Significant prognostic factors are shown in Table 3 and Figure 3.

**Table 3:** Univariate analysis of prognostic factors in patients treated with RFA after first-line treatment with TACE

**Variable**	**No. of patients (%)**	**OS (months)**	**P value**
Age (years)			
≥60	18 (40)	34	0.708
<60	27 (60)	27	
Gender			
Male	39 (86.7)	34	0.388
Female	6 (13.3)	20	
AFP			
≥400 ng/ml	20 (44.4)	20	0.015
<400 ng/ml	25 (55.6)	43	
Lesion size			
≥50 mm	41 (91.1)	41	0.028
<50 mm	4 (8.9)	22	
Lesion number			
Single	10 (22.2)	29	0.888
Multiple	35 (77.8)	28	
ALT			
≥75 U/L	9 (20.0)	38	0.828
<75 U/L	36 (80.0)	42	
GGT			
≥100 U/L	21 (46.7)	34	0.869
<100 U/L	24 (53.3)	27	
ALB			
≥35 g/L	38 (84.4)	34	0.078
<35 g/L	7 (15.6)	20	
PT			
≥13 s	7 (15.6)	20	0.059
<13 s	38 (84.4)	34	

Multivariate analysis confirmed that tumor diameter (P = 0.045, hazard ratio [HR]: 0.228, 95% confidence interval [CI]: 0.054-0.968) and pretreatment serum AFP level (P = 0.024, HR: 2.239, 95% CI: 1.114-4.500) were independent predictors for survival (Table 4).

**Table 4:** Multivariate analysis of prognostic factors in patients with RFA after first-line treatment with TACE

**Variable**	**P value**	**RR**	**95% CI**
AFP	0.024	2.239	1.114-4.500
Tumor size	0.045	0.228	0.054-0.968
Tumor number	0.565	0.775	0.325-1.848

## Discussion

RFA has been widely used as a curative therapy for small HCC. The overall survival of patients with small HCC undergoing RFA is similar to that of patients receiving surgical Resection [8-10,20,21]. In clinical practice it is rarely possible to achieve complete ablation for tumors larger than 5 cm because of the limitation of the ablation zone [22,23]. For patients with tumors beyond the Milan criteria, surgical treatment could be another curative option. If the tumor cannot be completely removed, palliative TACE treatment is the main treatment of choice. However, the long-term outcome for patients with unresectable HCC treated with TACE is unsatisfactory due to the inability to achieve complete tumor necrosis. Repeated TACE is often needed to completely eradicate the residual tumors, but its efficiency is limited and the rate of tumor recurrence or relapse after initial remission or stable disease is very high.

Several lines of evidence have indicated the feasibility and benefit of combination therapy of TACE and RFA. Buscarini et al. treated 14 HCC patients (with lesion size ranging from 3.8-6.8 cm, median 5.2 cm) with TACE followed by RFA. Their results suggested the possibility of treating large HCC with this procedure [24]. Lencioni et al. similarly reported a successful outcome (82%) among patients with HCC (lesion size ranging between 3.8 and 8.5 cm) who were treated with TACE prior to RFA [25]. Consistent with previous studies, the post-treatment CR rate of 76.2% and partial response rate of 23.8% in the present series indicate an encouraging benefit for patients with unresectable HCC beyond the Milan criteria.

RFA treatment following TACE has some advantages over TACE alone. Embolization during the TACE procedure can block arterial flow, which may reduce heat-sink effects during RFA thus increasing the volume of the zone of ablation and reducing the chance of tumor recurrence. TACE can also control or eliminate micro-metastasis, which cannot always be detected by ultrasonography, CT, or MRI. Thus, the addition of TACE may decrease the chance of micro-metastasis after RFA treatment in HCC patients with unresectable tumors beyond the Milan criteria.

With respect to prognostic factors correlated with local ablation efficiency, our results suggested that lesion diameter (< 50 mm vs. ≥ 50 mm) was statistically significant for predicting complete ablation (86.4% vs. 65%, P = 0.022). The presence of multiple lesions did not significantly affect complete ablation, although this might be because the number of nodules was confined to five in our study. It is difficult to completely destroy tumors larger than 5 cm by RFA despite multiple overlapping ablations; however, first-line TACE treatment might reduce the volume of viable tumor thus making complete ablation of the lesions possible. This treatment advantage was also proposed by Vogl, who suggested that repeated TACE might reduce the size of the treated lesions [26].

In this study, the 1-, 2-, and 3-year survival rates were 89%, 61%, and 43%, respectively. These rates are consistent with those of other studies. Veltri reported 1- and 2-year survival rates of 89.7% and 67.1% for TACE-RFA combined therapy for unresectable non-early HCC (size 30–80 mm, mean 48.9 mm) [27]. Similarly, Liao et al. [28] administrated TACE followed by RFA in the treatment of unresectable HCC (size 30–120 mm, mean 58.9 mm) with 1-, 2- and 3-year survival rates of 84%, 57%, and 38%, respectively. Our multivariate analysis showed that tumor size and serum AFP level before treatment were prognostic factors for overall survival. A high pretreatment level of AFP has previously been reported to be associated with poorer survival, reflecting not only tumor cell proliferation but also active disease with continuous necrosis and regeneration [29,30].

We found that TACE-RFA combined therapy had a low rate of major complications. No permanent adverse sequelae or treatment-related deaths were observed. Thus, combination therapy of TACE followed by RFA appears to be relatively safe.

## Conclusion

In conclusion, our results indicate that combination therapy of TACE with RFA is safe and effective, and can achieve a favorable long-term outcome in unresectable HCC beyond the Milan criteria. The major limitation of this study was its retrospective nature and non-randomized design. Further prospective randomized studies are warranted to confirm the efficacy of this promising combination therapy.

## Abbreviations

5-FU: 5-fluorouracil; AFP: Alpha-fetoprotein; CR: Complete response; CT: Computed tomography; HCC: Hepatocellular carcinoma; MRI: Magnetic resonance imaging; OS: Overall survival; RFA: Radiofrequency ablation; TACE: Transcatheter arterial chemoembolization.

## Competing interests

The authors declare that they have no competing interests.

## Authors’ contribution

LZ did literature search and wrote the paper; XY and XYX followed the patients; YHG, BHZ, YC, NLG, YHW and S LY carried out the TACE and RFA treatment; JBZ carried out the data analysis; ZGR revised the manuscript. All authors read and approved the final manuscript.

## Pre-publication history

The pre-publication history for this paper can be accessed here:

http://www.biomedcentral.com/1471-230X/14/11/prepub
